# Cardiometabolic multimorbidity and frailty in middle-aged and older adults: a cross-nationally harmonized study

**DOI:** 10.3389/fpubh.2025.1565682

**Published:** 2025-04-16

**Authors:** Kunyan Zhou, Ao Wang, Ke Yi

**Affiliations:** Key Laboratory of Obstetrics and Gynecologic and Pediatric Diseases and Birth Defects of Ministry of Education, West China Second University Hospital, Sichuan University, Chengdu, China

**Keywords:** multicohort cardiometabolic diseases, frailty index, diabetes, heart disease, stroke, middle-aged and older people

## Abstract

**Background:**

Cardiometabolic diseases are prevalent among ageing populations and have a close association with frailty. However, the cumulative impact multiple cardiometabolic diseases have on frailty remains underexplored.

**Methods:**

This study used data from four international cohorts – HRS, CHARLS, ELSA and SHARE – to examine the correlation between frailty and cardiometabolic diseases (CMD). The frailty index was used for assessing frailty and statistical analyses were performed as a means of analysing the correlation between the number of cardiometabolic conditions and frailty severity. Linear regression models were employed to evaluate the associations between CMD and frailty severity.

**Results:**

The study found that as the number of cardiometabolic diseases increased, the frailty index rose significantly [one disease, *β* = 7.80 (95% CI: 7.70 to 7.90) *p* < 0.05; two diseases, *β* = 17.92 (95% CI: 17.76 to 18.08) *p* < 0.05; three diseases, β = 28.79 (95% CI: 28.41 to 29.17) *p* < 0.05]. Stroke was found to have the most pronounced impact on frailty (*β* = 12.34 [95%CI 12.20 to 12.48] *p* < 0.05) and the coexistence of multiple conditions served to amplify the symptoms of frailty.

**Conclusion:**

This study highlights the compounded impact multiple cardiometabolic diseases have on frailty and also emphasizes the necessity for early intervention.

## Introduction

Cardiometabolic diseases (CMD), including stroke, diabetes and heart disease, have become increasingly prevalent among the ageing population (1). As people age, their risk of developing these chronic conditions increases significantly as a result of a combination of genetic, lifestyle and environmental factors (2–4). Diabetes, which is characterized by impaired glucose regulation, is particularly common in older adults and is often compounded by obesity and physical inactivity (5). CMDs, which involve heart failure, hypertension and coronary artery diseases are prevalent in ageing populations and contribute to increased morbidity and mortality (6). Similarly, stroke incidence increases with age and is driven by factors including hypertension, atherosclerosis and atrial fibrillation (7). These diseases are highly prevalent and also frequently coexist, which compounds the health burden of older adults, increasing the risk of disability and frailty and lowering their quality of life (8, 9).

In older people, frailty is a prevalent and debilitating condition that is marked by an increased susceptibility to unfavourable health problems and reduced physiological reserves (10). The key factors that contribute to frailty include ageing, chronic diseases, physical inactivity, malnutrition and psychological stress (11). These factors serve to create a vicious cycle, with frailty negatively impacting multiple health domains, including functional impairment, reduced mobility and a loss of independence (12, 13). As frailty progresses, life quality is significantly diminished by impaired mental health, physical capacities and social interaction (14, 15). In addition, frailty has a close association with higher risks of hospitalization, disability and mortality (16, 17).

Cardiometabolic diseases such as stroke, diabetes and heart disease make a significant contribution to this decline in physical function by impairing critical physiological processes (18, 19). These conditions lead to vascular damage, insulin resistance, muscle wasting and neurodegeneration, which all undermine physical capacity and increase frailty risk (20–22). Frailty becomes more pronounced as the number of cardiometabolic diseases increases, with a progressive decline in physical strength and overall resilience (23).

Cardiometabolic diseases are highly prevalent among older populations and have been shown to be strongly associated with frailty (24–26). However, existing research has predominantly focused on the impact of single cardiometabolic conditions on frailty, with limited exploration of the cumulative effects of comorbid cardiometabolic diseases (27). Moreover, most studies to date are based on data from a single region, lacking cross-national validation. This study leverages four international longitudinal cohorts (HRS, CHARLS, ELSA, and SHARE) to systematically examine how multiple cardiometabolic diseases interact to influence frailty, while also investigating the moderating effects of gender and age in this relationship.

Although previous studies have examined the associations between CMD or cardiometabolic multimorbidity (CMM) and outcomes such as depression, cognitive decline, and disability (28–30), these studies primarily focus on individual health outcomes rather than the broader construct of frailty. The uniqueness of our study lies in its comprehensive approach to assessing how the cumulative impact of CMD exacerbates frailty. By constructing a Frailty Index, we evaluate an individual’s health holistically, considering physiological, psychological, and functional domains. This approach moves beyond the limitations of studying isolated diseases or impairments and provides a more integrated understanding of health outcomes. Our research fills a significant gap in the current literature and offers critical insights that can inform the development of public health policies and targeted intervention strategies.

## Methods

### Study design and population

This integrated multicohort analysis used data from four international longitudinal cohorts targeting older and middle-aged people: the China Health and Retirement Longitudinal Study (CHARLS), the Health and Retirement Study (HRS), the Survey of Health, Ageing and Retirement in Europe (SHARE) and the English Longitudinal Study of Ageing (ELSA). The study used data from approximately overlapping time frames: HRS covers waves 10 to 15 (2010–2020), CHARLS includes waves 1 to 4 (2011–2018), ELSA encompasses waves 7 to 9 (2014–2018) and SHARE spans waves 4 to 7 (2011–2017) (Supplementary Table 2). Participants needed to be 50 years of age or older to be included and exclusions were applied to individuals with missing data relating to cardiometabolic diseases or frailty index. The study received ethical approval from the relevant committees for each study and participants were recruited after providing written informed consent.

### Exposure assessment

In the context of cardiometabolic diseases, the study focused on diabetes, heart disease and stroke as these conditions all have the potential to exacerbate frailty. The presence of these diseases was determined by face-to-face interviews between researchers and participants and supplemented by self-reported medical histories that were obtained from structured questionnaires. The cardiometabolic disease status of participants was classified on the basis of the total number of conditions they had (i.e., diabetes, heart disease or stroke). Participants were then categorized into three groups: those without any cardiometabolic disease, those with a single cardiometabolic condition and those who exhibited cardiometabolic multimorbidity (with two or more coexisting conditions).

### Outcome assessment

The frailty index, which quantifies the cumulative burden of age-related health deficits, was used to assess frailty (31). In accordance with previous studies, items that could be consistently applied across all four cohorts were selected (32, 33). After data screening, 30 items from the CHARLS, HRS, ELSA and SHARE surveys were included to construct the frailty index. These items encompassed self-reported health status, a range of chronic disorders, depression, functional limitations and cognitive impairment (Supplementary Table 3). Most of the items were dichotomised based on established cut-off values, with a score of 0 denoting the lack of a deficit and a score of 1 denoting its existence. Self-reported measures of general vision, hearing, health status and cognition were scored on a scale from 0 to 1, with higher scores indicating more serious deficits. The frailty index was computed by summing the deficits present in each individual, dividing by 30 and then multiplying by 100 for this study. Therefore, the frailty index was theoretically a continuous variable with a range from 0 to 100. Participants who missed any of the 30 items in the four databases were excluded from the process of frailty index calculation.

### Data collection

The following information was collected for this study: (i) demographic information: this consisted of marital status, sex, age and educational attainment. Three groups were created based on educational attainment: lower secondary education or below, upper secondary and higher than upper secondary. Marital status was divided into married and other marital statuses (such as separated, single, widowed and divorced). (ii) Lifestyle information: information was collected related to drinking and smoking patterns. Physical activity was defined as engaging in moderate or vigorous exercise at least once per week. (iii) Anthropometric measurements: body mass index (BMI). (iv) Medical history: information was gathered relating to the presence of hypertension, lung disease and cancer. For further details, please see Supplementary Table 4. In the data preprocessing of this study, we employed a complete case analysis method, including only samples with complete data for analysis. Variables or samples with a high proportion of missing values were subject to rigorous screening and exclusion to ensure data integrity.

### Statistical analysis

For continuous variables with a normal distribution, the data was presented as mean ± standard deviation (SD) and group differences were assessed through the use of ANOVA. Categorical variables were expressed as numerical values (percentages) and intergroup differences were analysed using Pearson’s chi-square test.

In the assessment of cardiometabolic diseases, we not only considered the classification based on the number of cardiometabolic diseases but also further analyzed the impact of different cardiometabolic diseases combinations on the frailty index. Specifically, we categorized the subjects into the following groups based on whether they had diabetes, heart disease, or stroke: (1) diabetes only, (2) heart disease only, (3) stroke only, (4) both diabetes and heart disease, (5) both diabetes and stroke, (6) both heart disease and stroke, and (7) all three diseases. Linear regression models were used to assess the relationship between cardiometabolic diseases and frailty index. Multiple models were constructed, each adjusting for a different set of covariates to provide a more detailed understanding of their impact on the observed association. Model 1 included no adjustments, Model 2 adjusted for age and gender, and Model 3 adjusted for age, gender, marital status, education, obesity, hypertension, cancer, lung disease, physical activity, and current smoking and drinking status.

Subgroup analyses were conducted to assess whether the association between CMD and the frailty index varies in strength across different populations. Participants were divided into different subgroups based on their gender, age (less than 65 years vs. more than 65 years), marital status, drinking and smoking habits and physical activity. MSTATA software^1^ and R software (version 4.3.1) were used for all of the statistical analyses. A two-sided *p*-value of below 0.05 was considered to be statistically significant.

### Results

**Figure 1:**
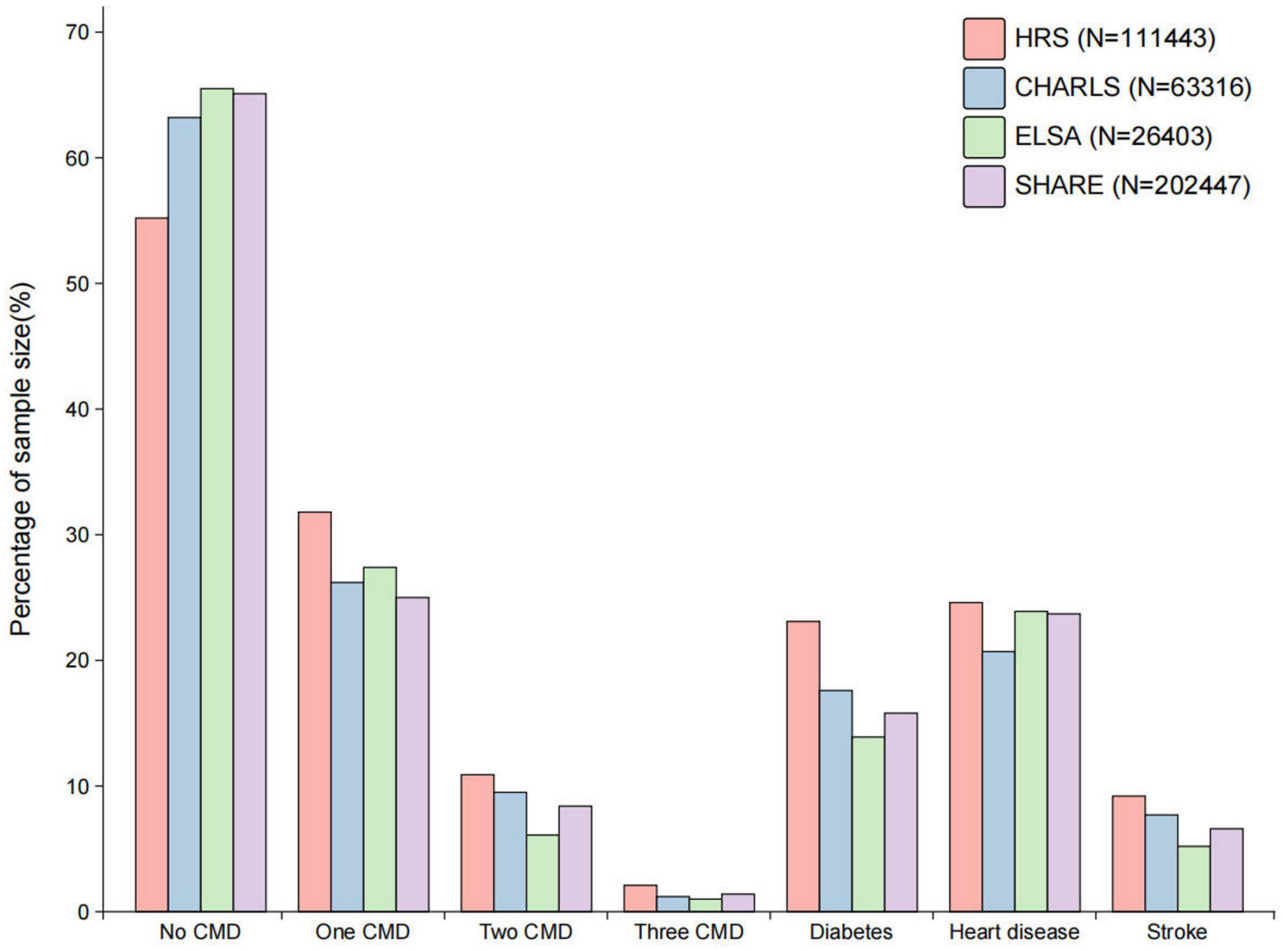
The distribution of cardiometabolic diseases across the various databases. CMD, cardiometabolic diseases; HRS, Health and Retirement Study; CHARLS, China Health and Retirement Longitudinal Study; ELSA, English Longitudinal Study of Ageing; SHARE, Survey of Health, Ageing and Retirement in Europe.

The flowchart for the investigation population screening procedure in HRS, CHARLS, ELSA and SHARE can be seen in Supplementary Figures 1–4. There was a total of 403,609 participants and their mean age was 66.7 (SD 10.3) years. 224,867 (55.7%) of the participants in the final analytic cohort were female and 178,740 (44.3%) were male. Figure 1 shows the distribution of cardiometabolic diseases in HRS, CHARLS, ELSA and SHARE. 257,525 (63.8%) of the participants were free from any cardiometabolic diseases, while 146,084 (36.2%) exhibited at least one form of cardiometabolic disease. The frailty index for participants without cardiometabolic diseases was 14.2 (SD 11.2). In contrast, those with a single cardiometabolic disease had a higher frailty index of 25.1 (SD 14.4), while those with two or more cardiometabolic diseases exhibited an even further increase, with a frailty index of 37.5 (SD 17.6) (Table 1). The baseline table classified by diabetes, heart disease and stroke is shown in Supplementary Table 5. The distribution of the frailty index across the various databases can be seen in Supplementary Figure 5.

**Table 1:** Characteristics of participants by cardiometabolic disease status.

	No cardiometabolic diseases (*n* = 257,525)	One cardiometabolic disease (*n* = 106,822)	Cardiometabolic disease multimorbidity (*n* = 39,262)
Frailty	14.23 ± 11.23	25.11 ± 14.44	37.54 ± 17.57
Age	64.56 ± 9.66	69.65 ± 10.32	72.29 ± 10.04
Missing	207	54	13
Gender			
Female	147,173 (57.1%)	57,569 (53.89%)	20,125 (51.3%)
Male	110,350 (42.9%)	49,253 (46.11%)	19,137 (48.7%)
Missing	2	0	0
Educational attainment			
Lower secondary education or below	103,142 (40.3%)	45,186 (42.56%)	17,720 (45.3%)
Upper secondary	100,074 (39.1%)	43,229 (40.71%)	15,957 (40.8%)
Higher than upper secondary	52,626 (20.6%)	17,765 (16.73%)	5,436 (13.9%)
Missing	1,683	642	149
Marital status			
Other	81,658 (31.7%)	40,097 (37.54%)	16,584 (42.2%)
Married	175,867 (68.3%)	66,725 (62.46%)	22,678 (57.8%)
Obesity			
Underweight or normal (<25 kg/m^2^)	100,340 (39.7%)	30,684 (29.35%)	9,440 (24.7%)
Overweight (25–29.9 kg/m^2^)	100,935 (39.9%)	42,118 (40.29%)	14,477 (37.9%)
Obesity (≥30 kg/m^2^)	51,466 (20.4%)	31,731 (30.36%)	14,308 (37.4%)
Missing	4,784	2,289	1,037
Hypertension	96,560 (37.9%)	69,284 (64.90%)	31,676 (80.7%)
Missing	2,750	67	18
Cancer	20,524 (8.1%)	12,897 (12.08%)	6,059 (15.4%)
Missing	2,924	31	9
Lung disease	18,585 (7.3%)	14,042 (13.15%)	7,787 (19.8%)
Missing	2,875	39	9
Current drinking	42,032 (16.3%)	12,250 (11.47%)	3,004 (7.7%)
Missing	219	18	5
Current smoking	62,898 (24.6%)	21,655 (20.36%)	7,890 (20.2%)
Missing	1,420	481	251
Physical activity	211,229 (82.0%)	75,079 (70.28%)	21,712 (55.3%)
Missing	5	0	1

Participants without cardiometabolic diseases were used as the reference group in this study. Among those with a single cardiometabolic condition, a modest yet statistically significant increase in frailty index was observed [unadjusted *β* = 7.80 (95% CI: 7.70 to 7.90)]. In addition, a more rapid increase in the frailty index was observed with the increasing number of cardiometabolic conditions. More specifically, the frailty index increased significantly in individuals with two cardiometabolic diseases [unadjusted *β* = 17.92 (95% CI: 17.76 to 18.08)] and more markedly in those with three cardiometabolic diseases [unadjusted β = 28.79 (95% CI: 28.41 to 29.17)]. Model 2 made adjustments for both gender and age, while Model 1 was an unadjusted model. Several covariates, including obesity, cancer, lifestyle factors and educational attainment, were taken into consideration by Model 3 (Table 2). These patterns remained significant even after adjustments for covariates were made.

**Table 2:** Associations between cardiometabolic multimorbidity status and frailty.

	Model 1	Model 2	Model 3
	Beta (95% CI)	Beta (95% CI)	Beta (95% CI)
No cardiometabolic diseases	Ref	Ref	Ref
One cardiometabolic disease	7.80 (7.70, 7.90)	5.89 (5.79, 5.99)	3.50 (3.42, 3.58)
Two cardiometabolic diseases	17.92 (17.76, 18.08)	15.29 (15.15, 15.44)	10.46 (10.31, 10.57)
Three cardiometabolic diseases	28.79 (28.41, 29.17)	25.51 (25.15, 25.86)	17.96 (17.65, 18.26)

Model 1: no covariates were adjusted. Model 2: adjusted for Age and Gender. Model 3: adjusted for Age, Gender, Marital status, Educational attainment, Obesity, Hypertension, Cancer, Lung disease, Current drinking, Current smoking, and Physical activity.

**Figure 2:**
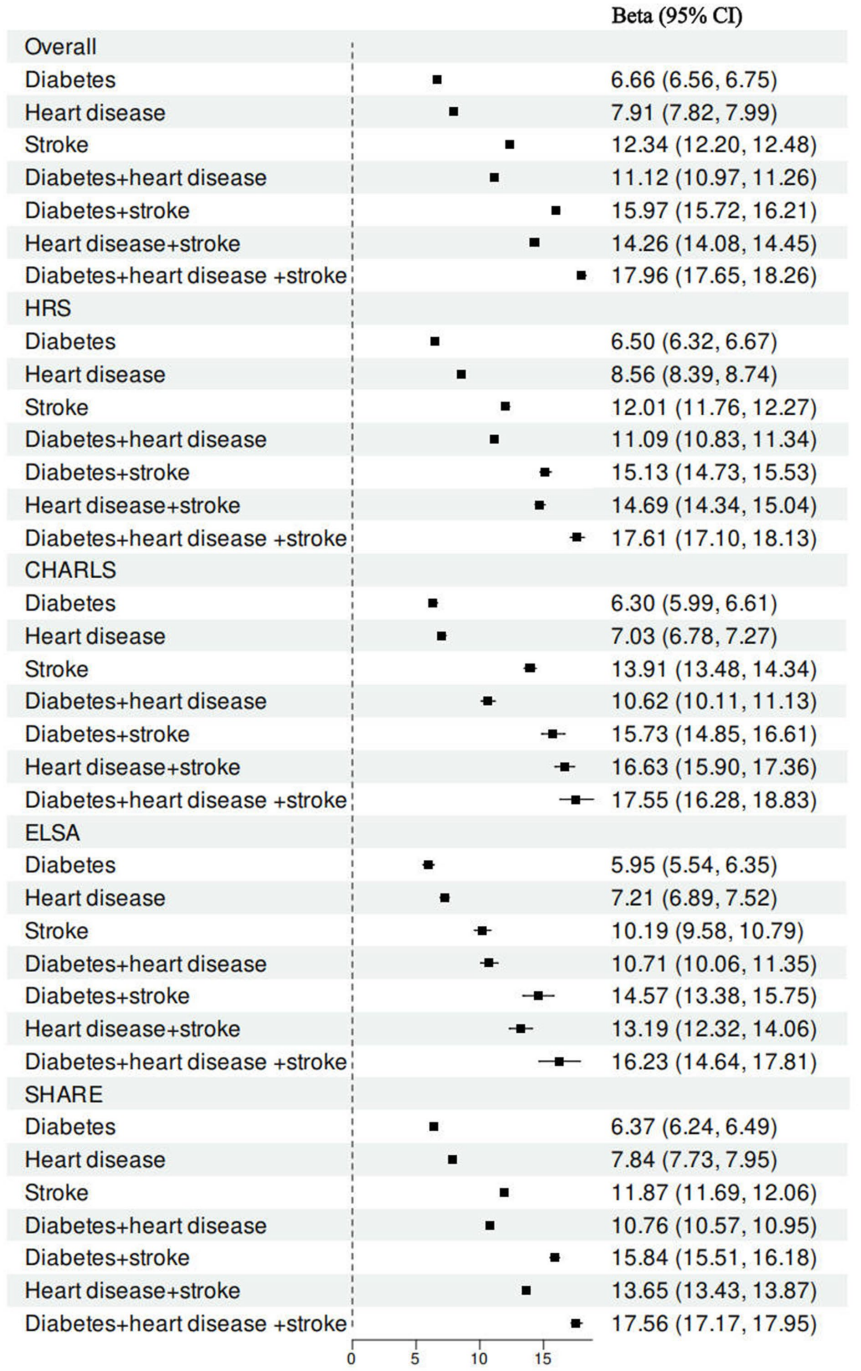
Associations between cardiometabolic disease status and frailty index by specific combination of individual cardiometabolic diseases. All models were adjusted for age gender, marital status, educational attainment, obesity, hypertension, cancer, lung disease, current drinking, current smoking, and physical activity.

Analysis of the relationships between specific combinations of individual cardiometabolic diseases and frailty indicated that stroke had the most significant effect on the frailty index of all the single cardiometabolic conditions (Figure 2). Among combinations of two coexisting cardiometabolic diseases, the pair of diabetes and stroke was found to be most strongly associated with a more rapid increase in the frailty index. Furthermore, in comparison to the presence of one or two cardiometabolic diseases, the coexistence of three cardiometabolic diseases was found to have a significant association with a more substantial increase in the frailty index (Supplementary Tables 6–9).

**Figure 3:**
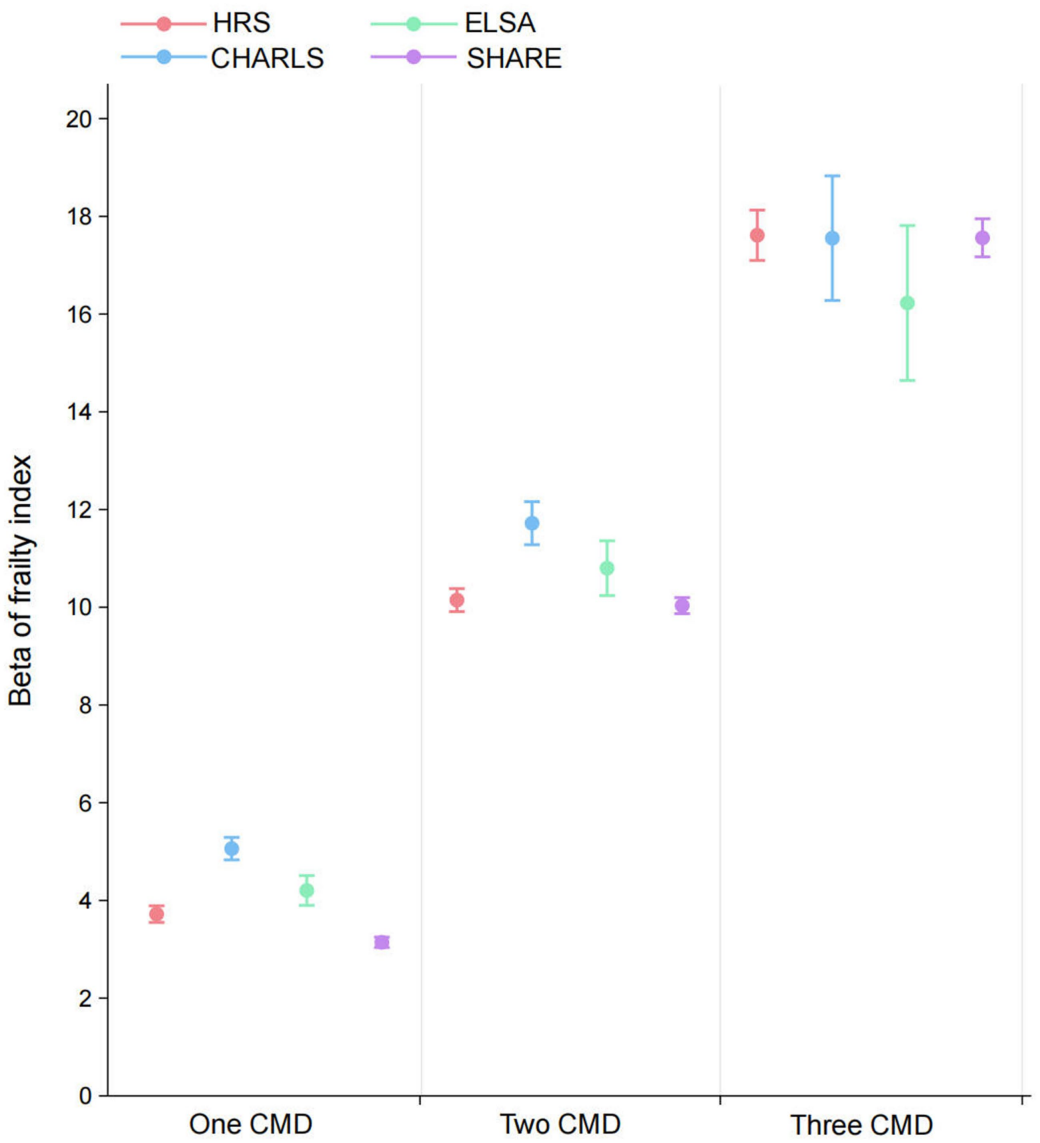
Associations between cardiometabolic multimorbidity status and frailty index in HRS, CHARLS, ELSA, and SHARE. All models were adjusted for age gender, marital status, educational attainment, obesity, hypertension, cancer, lung disease, current drinking, current smoking, and physical activity. CMD, cardiometabolic diseases; HRS, Health and Retirement Study; CHARLS, China Health and Retirement Longitudinal Study; ELSA, English Longitudinal Study of Ageing; SHARE, Survey of Health, Ageing and Retirement in Europe.

In addition to the overall analysis, the association between the prevalence of cardiometabolic diseases and frailty across the four datasets was examined. A dose–response correlation between the number of cardiometabolic diseases and the frailty index was found in the HRS, with *β* values increasing progressively as the number of comorbidities increased [single cardiometabolic disease: β = 3.72 (95% CI: 3.55 to 3.89), two cardiometabolic diseases: β = 10.15 (95% CI: 9.91 to 10.38), three cardiometabolic diseases: β = 17.61 (95% CI: 17.10 to 18.13)]. The CHARLS, ELSA and SHARE datasets all exhibited a similar trend (Figure 3).

In the subgroup analysis, we employed interaction tests (P for interaction) to assess the differences in the strength of the association between CMD and the frailty index across different subgroups. The results showed that the interaction effects of age (P for interaction <0.01) and gender (P for interaction <0.01) were statistically significant, while the interaction effects of current drinking (P for interaction = 0.912), current smoking (P for interaction = 0.993), and physical activity (P for interaction = 0.134) were not significant. Specifically, in the older population (age ≥ 65), the association between CMD and the frailty index was stronger (*β* = 6.74, 95% CI: 6.62–6.86, *p* < 0.01), whereas in the younger population (age < 65), the association was weaker (β = 5.00, 95% CI: 4.87–5.14, *p* < 0.01). Similarly, in the female subgroup, the association was more pronounced (β = 7.37, 95% CI: 7.24–7.51, *p* < 0.01), while in the male subgroup, the association was weaker (β = 5.76, 95% CI: 5.63–5.88, *p* < 0.01). These results suggest that gender and age may be important moderating factors in the relationship between CMD and the frailty index, while drinking, smoking, and physical activity do not significantly impact this relationship (Supplementary Figure 6).

## Discussion

This study has summarized the prevalence of cardiometabolic diseases across four international cohorts (HRS, CHARLS, ELSA and SHARE) and explored the relationship between cardiometabolic multimorbidity and frailty. The findings revealed a significant increase in frailty index as the number of cardiometabolic conditions increased, with greater multimorbidity having a significant correlation with more severe frailty. More specifically, those with a single cardiometabolic disease already had a higher frailty index, while those with two or more conditions had a more pronounced increase in frailty. Further analysis served to demonstrate a distinct dose–response correlation between the frequency of cardiometabolic diseases and the frailty index, particularly in individuals with stroke, where the increase in frailty was particularly marked. The coexistence of diabetes and stroke was found to have the most significant impact on frailty, potentially due to the compounded effect these conditions have on physical decline and overall health burden. These findings serve to highlight the substantial impact cardiometabolic multimorbidity has on frailty in older populations, which shows that there is a need for clinical interventions to address multiple comorbidities in older adult patients.

Cardiometabolic diseases, which include diabetes, heart disease and stroke, share several overlapping pathophysiological processes that make a collective contribution to the exacerbation of frailty (34). One of the primary mechanisms is chronic inflammation, which is a common feature with all of these conditions (35, 36). In diabetes, insulin resistance promotes a pro-inflammatory state (37), while heart disease is associated with systemic inflammation that is driven by endothelial dysfunction and atherosclerosis (38, 39). Stroke leads to neuroinflammation as a result of cerebral ischemia and neuronal injury, particularly in its chronic phase (40, 41). The systemic elevation of inflammatory markers, such as tumour necrosis factor-*α* (TNF-α) and C-reactive protein (CRP) (42), is important in the pathogenesis of frailty as it contributes to vascular damage (43), muscle atrophy (44) and diminished functional capacity (45), thereby accelerating frailty progression.

Oxidative stress is another shared pathological process that plays a significant role in the cellular damage that is observed in these diseases (46). In diabetes, hyperglycaemia leads to an excess of ROS, which causes damage to cellular structures, including endothelial cells, thereby exacerbating cardiovascular complications (47). Similarly, in heart disease, persistent heart failure contributes to impaired tissue oxygenation, which further amplifies oxidative stress (48). After a stroke, cerebral ischemia induces mitochondrial dysfunction, increasing ROS production and damaging both neuronal and muscle tissues (49, 50). The cumulative oxidative damage in skeletal muscles results in muscle wasting and weakness, which are key components of frailty (51, 52).

Autonomic dysfunction is a critical common pathway in cardiometabolic diseases, which affects the neuro-muscular coordination that is necessary for maintaining physical strength and stability (53). Diabetic neuropathy and the disruption of central nervous system control following a stroke impair motor coordination, which leads to reduced physical performance and mobility (54, 55). These mechanisms act synergistically and this results in more severe frailty in those with multiple cardiometabolic comorbidities.

In comparison to previous studies, the results of this study are largely consistent and demonstrate broader applicability. Gao et al. identified a significant association between cardiometabolic multimorbidity, frailty and healthcare utilization and highlighted that the presence of multiple cardiometabolic diseases increases the incidence of frailty and healthcare expenditure (23). In this study, the findings from datasets from the United States, England and Europe similarly indicate a clear dose–response relationship between cardiometabolic multimorbidity and frailty. More specifically, as the number of cardiometabolic conditions increases, the severity of frailty and the corresponding healthcare demands both exhibit a substantial increase.

There is a notable alignment when comparing the findings of Tang et al. with the results from the subgroup analysis of this study, particularly regarding gender differences. Tang et al. found women to have a higher frailty index than men, particularly in the presence of cardiometabolic diseases, such as stroke (56). The subgroup analysis in this study found that the association between CMD and the frailty index was significantly stronger in females than in males. Several factors may have contributed to these differences, such as hormonal variations, particularly the decline in oestrogen post-menopause, which accelerates muscle loss and increases frailty risk in women (57). In addition, it is typical for women to experience a higher burden of chronic illnesses, including CVD and osteoporosis, which further exacerbates frailty (58).

Furthermore, our study was compared with the study by Luo et al. (59), which explored the relationship between multimorbidity and frailty transitions in USA, finding that multimorbidity significantly increased the risk of frailty deterioration, with distinct patterns affecting frailty transitions. In contrast, our research utilized data from four international cohorts and focused on cardiometabolic diseases, revealing that stroke had the most pronounced impact on frailty. This study not only corroborates previous findings but also provides novel insights, emphasizing the cumulative impact of multiple cardiometabolic conditions on frailty, thus offering a new perspective for clinical intervention strategies.

Our findings suggest that CMD significantly increases the risk of frailty, particularly among older adults and women. Therefore, public health policies should place greater emphasis on high-risk populations, such as through regular health screenings and personalized health management, to identify and intervene in potential frailty risks at an early stage (15, 60). From a disease prevention perspective, promoting healthy lifestyles should be incorporated into national and regional public health programs. For example, optimizing nutritional guidance, encouraging regular physical activity, and managing chronic disease risk factors can effectively reduce the incidence of frailty.

From a healthcare policy standpoint, our study provides a foundation for optimizing chronic disease management. As CMD patients often require long-term, multidisciplinary medical support, healthcare systems should strengthen the capacity of primary healthcare institutions in managing chronic diseases, such as by offering integrated health management services, reducing repeated hospitalizations, and minimizing medical resource waste. These measures will not only improve patient health outcomes but also reduce the burden on the healthcare system and enhance resource utilization efficiency.

The strengths of this study include the use of multinational data, its large sample size and the use of harmonized standards across four international cohorts, which served to significantly enhance the generalisability and representativeness of the findings. By including diverse populations from different geographical regions, the study provided a robust analysis of cardiometabolic multimorbidity and frailty, which allowed for more accurate comparisons in a variety of healthcare systems and cultural contexts.

However, the study also has a number of limitations that must be noted. Firstly, some health conditions and lifestyle factors were based on self-reported data, which may present biases such as social desirability bias and recall bias, thereby potentially compromising the accuracy of the information that was supplied. Although our study employed complete case analysis to ensure the robustness of the data, we recognize that methods such as multiple imputation could further enhance data utilization and reduce potential selection bias. Secondly, although this study utilized multiple international cohorts (HRS, CHARLS, ELSA, SHARE), offering a broad sample representation, we acknowledge that there are methodological differences in the data collection processes across these cohorts. For instance, variations in disease diagnosis, health indicator measurements, and survey design may introduce certain biases during data integration. While we employed linear regression models to examine the relationship between cardiometabolic diseases and frailty, the statistical analysis may still be influenced by unmeasured variables, such as dietary habits and socioeconomic factors, which could potentially confound the results. Future research could consider employing more rigorous standardization methods or statistical strategies like propensity score matching to reduce inter-cohort biases and enhance the robustness of the findings. In addition, cross-sectional data was used in this investigation, which served to restrict the capacity to draw clear causal inferences between cardiometabolic multimorbidity and frailty. Furthermore, although this study has employed multilevel regression models and subgroup analyses to ensure the accuracy and robustness of the analyses, we acknowledge that further statistical techniques, such as more advanced Bayesian methods or machine learning approaches, could offer a more comprehensive assessment of the relationship between cardiometabolic diseases and the frailty index. However, due to constraints in time and resources, we have not yet conducted more extensive statistical expansions. In future research, we intend to incorporate additional statistical methodologies to further enhance the depth and precision of the study.

## Conclusion

This study demonstrates a significant dose–response relationship between cardiometabolic multimorbidity and frailty, with stroke having the most pronounced impact. Older adults and women, in particular, are more susceptible to the exacerbation of frailty due to the influence of multiple cardiometabolic conditions. These findings underscore the importance of implementing early intervention strategies targeting the aging population to mitigate the risk of frailty.

## Data Availability

Publicly available datasets were analyzed in this study. This data can be found here: CHARLS (https://charls.pku.edu.cn/en), ELSA (https://www.elsa-project.ac.uk/), SHARE (https://share-eric.eu/), and HRS (https://hrs.isr.umich.edu/). The original contributions presented in the study are included in the article/Supplementary material. Further inquiries can be directed to the corresponding author.

## Footnotes

^1^ www.mstata.com
