# Supplementary material for: Cardiometabolic multimorbidity and frailty in middle-aged and older adults: a cross-nationally harmonized study
Source: Front Public Health. 2025 Apr 16;13:1565682. doi: 10.3389/fpubh.2025.1565682 (PMC12041071; doi:10.3389/fpubh.2025.1565682)
Supplement: Supplementary file 1 [file Data_Sheet_1.docx]

Supplementary content

[Supplementary Table 1. Regions for different studies. 2](#_Toc29489)

[Supplementary Table 2. Waves and years included in analyses from CHARLS, ELSA, HRS and SHARE. 3](#_Toc17816)

[Supplementary Figure 1. Flowchart of sample selection in HRS. 4](#_Toc2266)

[Supplementary Figure 2. Flowchart of sample selection in CHARLS. 5](#_Toc6497)

[Supplementary Figure 3. Flowchart of sample selection in ELSA. 6](#_Toc31705)

[Supplementary Figure 4. Flowchart of sample selection in SHARE. 7](#_Toc28540)

[Supplementary Table 3 The items used to construct the frailty index in HRS, CHARLS, SHARE,and ELSA. 8](#_Toc32223)

[Supplementary Table 4. Harmonized strategies for key variable in this study. 10](#_Toc10113)

[Supplementary Figure 5. The distribution of frailty index in HRS, CHARLS, ELSA, and SHARE. 11](#_Toc14890)

[Supplementary Table 5. Characteristics of participants by cardiometabolic diseases 12](#_Toc27055)

[Supplementary Figure 6. Subgroup analyses of the association between cardiometabolic diseases and frailty. 13](#_Toc8719)

[Supplementary Table 6. Association between cardiometabolic diseases and frailty in HRS 14](#_Toc27604)

[Supplementary Table 7. Association between cardiometabolic diseases and frailty in CHARLS 14](#_Toc7877)

[Supplementary Table 8. Association between cardiometabolic diseases and frailty in ELSA 15](#_Toc23141)

[Supplementary Table 9. Association between cardiometabolic diseases and frailty in SHARE 15](#_Toc23200)

[Supplementary Table 10. Association between one cardiometabolic disease and frailty in HRS, CHARLS, ELSA, and SHARE 16](#_Toc28492)

[Supplementary Table 11. Association between two cardiometabolic disease and frailty in HRS, CHARLS, ELSA, and SHARE 16](#_Toc9508)

[Supplementary Table 12. Association between three cardiometabolic disease and frailty in HRS, CHARLS, ELSA, and SHARE 17](#_Toc19514)

# Supplementary Table 1. Regions for different studies.

| Study | Regions | Country |
| --- | --- | --- |
| ELSA | Western Europe | England |
| CHARLS | East Asia | China |
| HRS | North America | United States |
| SHARE | North Europe | Denmark  Sweden  Finland |
|  | Western Europe | Austria  Belgium  France  Germany  Netherlands Switzerland  Ireland  Luxembourg |
|  | Southern Europe | Greece  Italy  Spain  Portugal  Bulgaria  Cyprus  Malta  Israel |
|  | Central Europe | Czech Republic  Poland  Hungary  Slovenia  Croatia  Slovakia |
|  | Eastern Europe | Estonia  Lithuania  Latvia  Romania |

# Supplementary Table 2. Waves and years included in analyses from CHARLS, ELSA, HRS and SHARE.

| Year | CHARLS | ELSA | SHARE | HRS |
| --- | --- | --- | --- | --- |
| 2010 |  |  | Wave 4 2010-2011 | Wave10 2010-2011 |
| 2011 | Wave 1 2011-2012 |  |  |  |
| 2012 |  |  | Wave 5 2012-2013 | Wave11 2012-2013 |
| 2013 | Wave 2 2013-2014 |  |  |  |
| 2014 |  | Wave 7 2014-2015 | Wave 6 2014-2015 | Wave12 2014-2015 |
| 2015 | Wave 3 2015-2016 |  |  |  |
| 2016 |  | Wave 8 2016-2017 | Wave 7 2016-2017 | Wave13 2016-2017 |
| 2017 |  |  |  |  |
| 2018 | Wave 4 2018-2019 | Wave 9 2018-2019 |  | Wave14 2018-2019 |
| 2019 |  |  |  |  |
| 2020 |  |  |  | Wave15 2020-2021 |
| 2021 |  |  |  |  |

Abbreviations: CHARLS: China Health and Retirement Longitudinal Study; ELSA: English Longitudinal Study of Ageing; HRS: Health and Retirement Study; SHARE: Survey of Health, Ageing and Retirement in Europe.

# Supplementary Figure 1. Flowchart of sample selection in HRS.


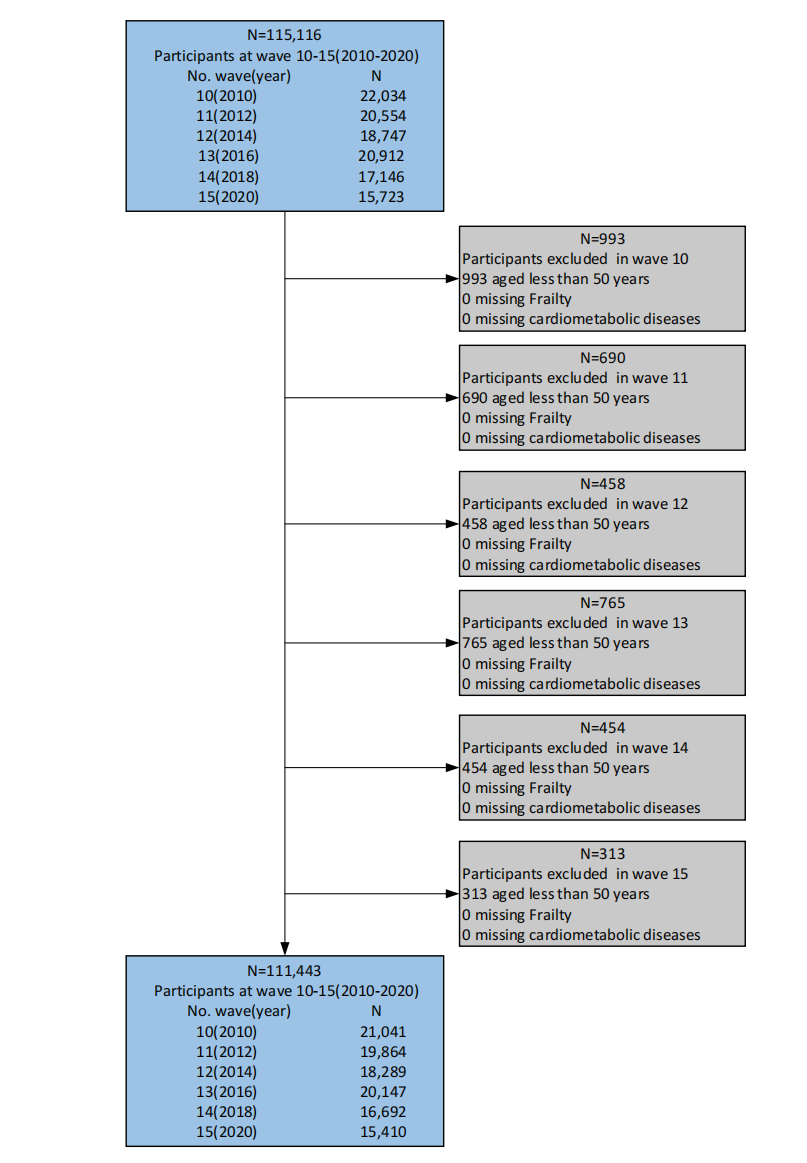


# Supplementary Figure 2. Flowchart of sample selection in CHARLS.


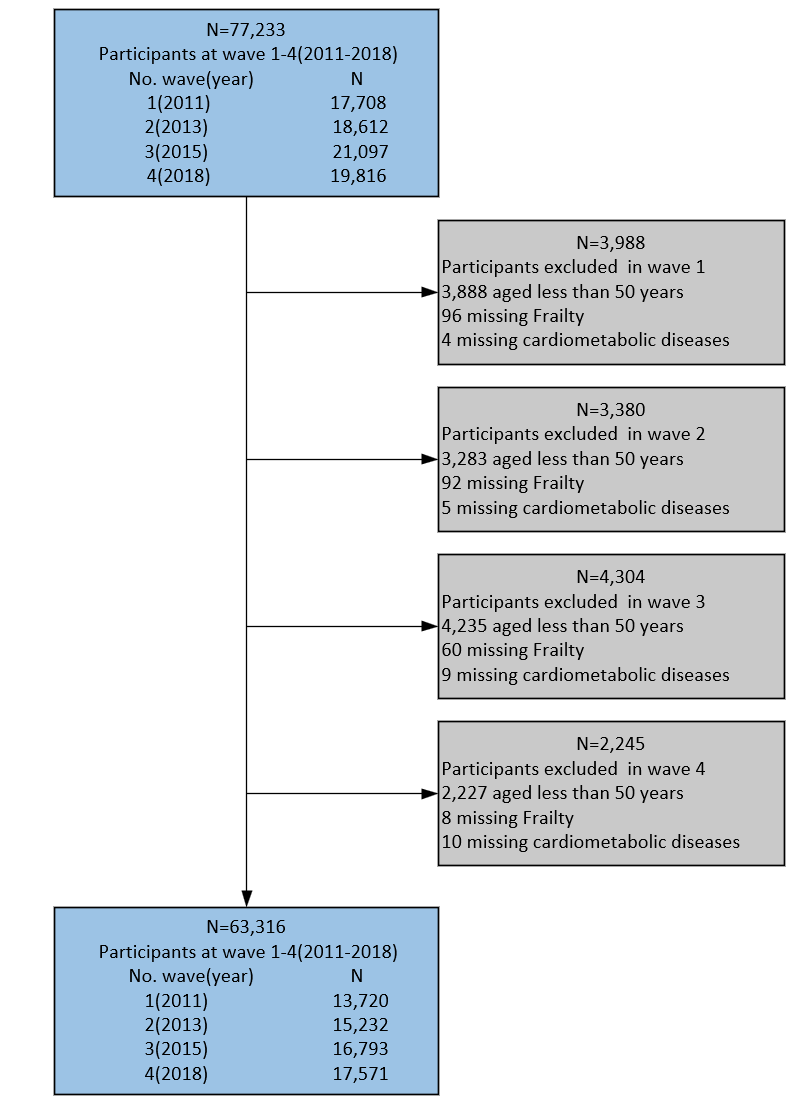


# Supplementary Figure 3. Flowchart of sample selection in ELSA.


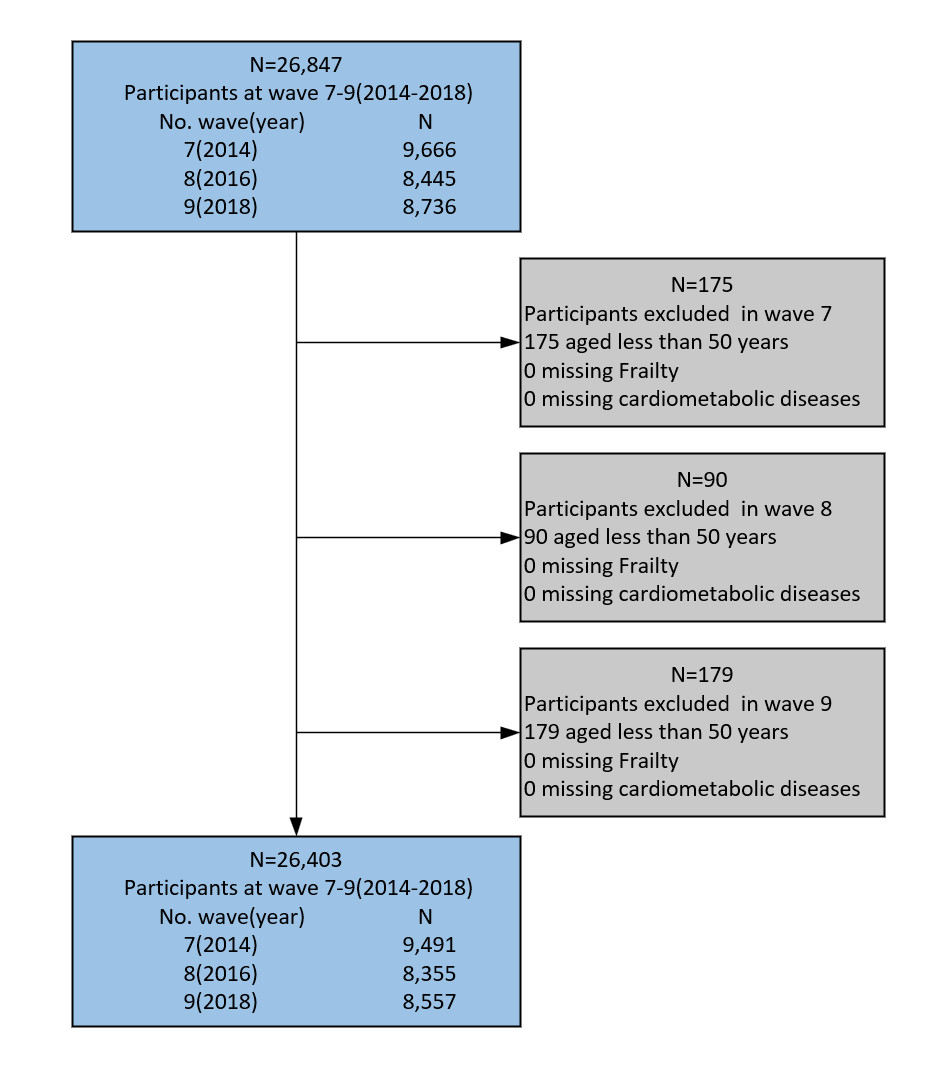


# Supplementary Figure 4. Flowchart of sample selection in SHARE.


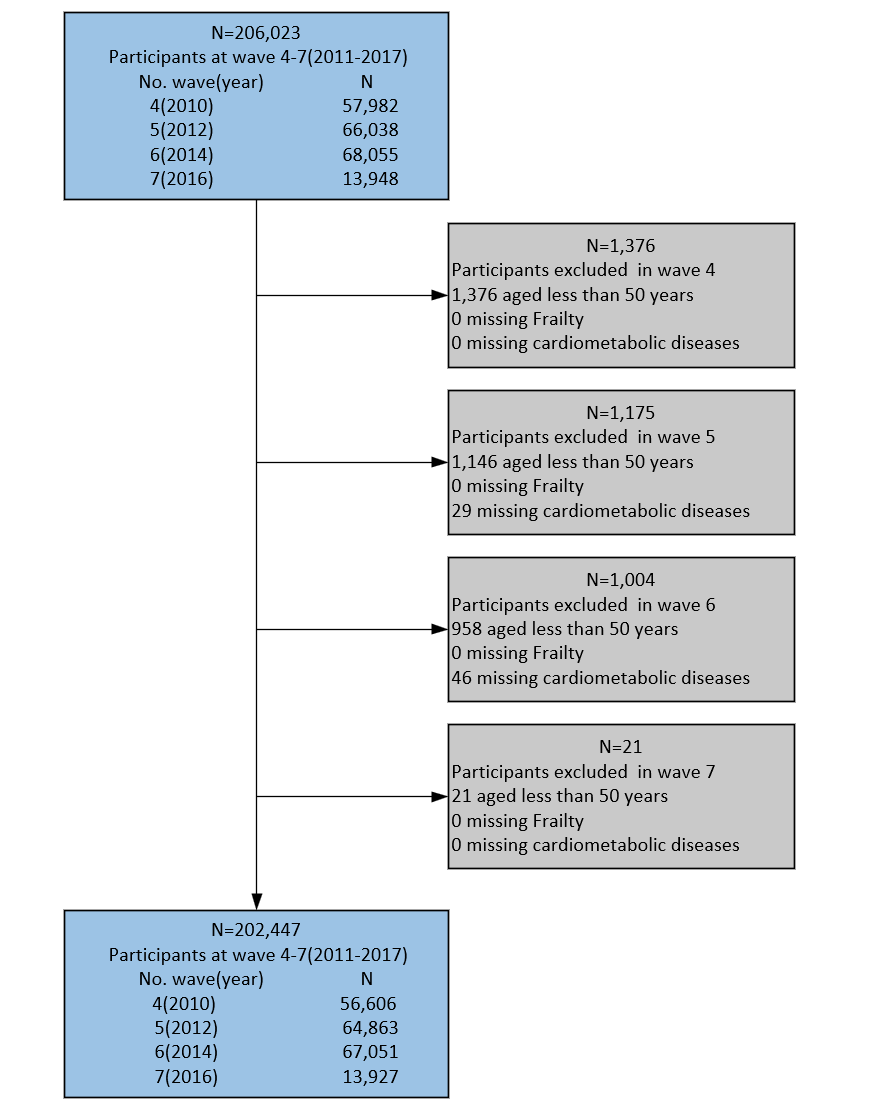


# Supplementary Table 3 The items used to construct the frailty index in HRS, CHARLS, SHARE,and ELSA.

| No | Description of the items | | | | Cut-off value |
| --- | --- | --- | --- | --- | --- |
|  | HRS (2012-2018) | CHARLS (2011-2018) | SHARE (2013-2019) | ELSA (2012-2018) |  |
| 1 | Self-reported diagnosed hypertension by doctors | | | | Yes=1, No=0 |
| 2 | Self-reported diagnosed diabetes by doctors | | | | Yes=1, No=0 |
| 3 | Self-reported diagnosed heart disease by doctors | | | | Yes=1, No=0 |
| 4 | Self-reported diagnosed stroke by doctors | | | | Yes=1, No=0 |
| 5 | Self-reported diagnosed cancer by doctors | | | | Yes=1, No=0 |
| 6 | Self-reported diagnosed arthritis by doctors | | | | Yes=1, No=0 |
| 7 | Self-reported diagnosed chronic lung disease by doctors | | | | Yes=1, No=0 |
| 8 | Self-reported diagnosed any emotional, nervous, or psychiatric problems by doctors | | | | Yes=1, No=0 |
| 9 | Self-reported diagnosed memory-related disease, including Alzheimer’s disease or dementia, organic brain senility, or other serious memory impairment | | | | Yes=1, No=0 |
| 10 | Self-reported general eyesight | Self-reported eyesight for seeing thing up close | | Self-reported general eyesight | Poor or blind=1, fair=0.75, good=0.5, very good=0.25, excellent=0 |
| 11 | Self-reported hearing while wearing hearing aid or as usual | | | | Poor or deaf=1, fair=0.75, good=0.5, very good=0.25, excellent=0 |
| 12 | Self-reported healthy status | | | | Poor=1, fair=0.75, good=0.5, very good=0.25, excellent=0 |
| 13 | BADL: Any difficulty in dressing | | | | Yes=1, No=0 |
| 14 | BADL: Any difficulty in bathing or showering | | | | Yes=1, No=0 |
| 15 | BADL: Any difficulty in eating | | | | Yes=1, No=0 |
| 16 | BADL: Any difficulty in getting in/out bed | | | | Yes=1, No=0 |
| 17 | BADL: Any difficulty in using the toilet | | | | Yes=1, No=0 |
| 18 | IADL: Any difficulty in managing money | | | | Yes=1, No=0 |
| 19 | IADL: Any difficulty in taking medications | | | | Yes=1, No=0 |
| 20 | IADL: Any difficulty in shopping | | | | Yes=1, No=0 |
| 21 | IADL: Any difficulty in preparing meals | | | | Yes=1, No=0 |
| 22 | Mobility: Any difficulty in walking 1 block | | | | Yes=1, No=0 |
| 23 | Mobility: Any difficulty in getting up from a chair after sitting for long periods | | | | Yes=1, No=0 |
| 24 | Mobility: Any difficulty in climbing several flights of stairs without resting | | | | Yes=1, No=0 |
| 25 | Mobility: Any difficulty in lifting or carrying weights over 10 pounds | | | | Yes=1, No=0 |
| 26 | Mobility: Any difficulty in picking up a coin from table | | | | Yes=1, No=0 |
| 27 | Mobility: Any difficulty in stooping kneeling or crouching | | | | Yes=1, No=0 |
| 28 | Mobility: Any difficulty in reaching arms above shoulder level | | | | Yes=1, No=0 |
| 29 | Depression: CESD-8 (ranging from 0 to 8) | Depression: CESD-10 (ranging from 0 to 30) | Depression: EURO (ranging from 0 to 12) | Depression: CESD-8 (ranging from 0 to 8) | CESD-8≥3, CESD-9≥5, CESD-10≥10, EURO≥4=1, CESD-8<3, CESD-9<5, CESD-10<10, EURO<4=0 |
| 30 | Cognition: (total score-(immediate and delayed word recall + date naming + serial 7’s))/total score | | | | Continuous, from 0 to 1 |

HRS, Health and Retirement Study; CHARLS, China Health and Retirement Longitudinal Study; SHARE, Survey of Health, Ageing and Retirement in Europe; ELSA, English Longitudinal Study of Ageing; CESD, Center for Epidemiologic Studies Depression Scale.

Cognition test consists of four components, including immediate and delayed word recall, date naming and serial 7’s. For word recall, participants are required to recite 10 words in HRS, CHARLS, SHARE, and ELSA. For date naming, participants are asked whether they could remember the date of that day (day of week, day of month, month, and year in HRS, CHARLS, SHARE, and ELSA). For serial 7’s, participants are required to make five calculations and answer how much is 100 minus 7. One point is given for each right answer, and the cognition score is calculated by the formula: (total score-(immediate and delayed word recall + date naming + serial 7’s))/total score. Total score refers to the theoretical maximum score of all tests. Hence, a higher cognition score indicated lower cognition function.

# Supplementary Table 4. Harmonized strategies for key variable in this study.

| **Variables** | **Harmonized values** | **Measurements in four studies** | | | |
| --- | --- | --- | --- | --- | --- |
|  |  | **ELSA** | **HRS** | **SHARE** | **CHARLS** |
| Stroke | No | The respondent reported having no stroke | | | |
|  | Yes | The respondent reported having stroke or receiving treatment for stroke | | | |
| Diabetes | No | The respondent reported having no diabetes | | | |
|  | Yes | The respondent reported having diabetes receiving treatment for diabetes | | | |
| Heart  disease | No | The respondent reported having no heart disease | | | |
|  | Yes | The respondent reported having heart disease receiving treatment for heart disease | | | |
| Kidney  disease | No | The respondent reported having no kidney disease | | | |
|  | Yes | The respondent reported having kidney disease | | | |
| Liver  disease | No | The respondent reported having no liver disease | | | |
|  | Yes | The respondent reported having liver disease | | | |
| Lung  disease | No | The respondent reported having no lung disease | | | |
|  | Yes | The respondent reported having lung disease | | | |
| Cancer | No | The respondent reported having no cancer | | | |
|  | Yes | The respondent reported having cancer | | | |
| Marital status | Married | Married | | | |
|  | Other | Other marital status (never married, separated, divorced, and widowed) | | | |
| Physical activity | Yes | Frequency of taking part in  vigorous/moderate physical activity: 1. everyday; 2. more than once a  week; 3. once a week; 4. one to three times a month | | | The number of days  of vigorous/moderate physical activity for at least 10 minutes |
|  | No | Hardly ever or never taking part in vigorous/moderate physical activity | | | No |
| Current smoking | No | No recent smoking | | | |
|  | Yes | Smoking at the present time | | | |
| Current drinking | No | The number of drinks per week (multiplying the number of days per week that alcohol was consumed the number of drinks per day): ≤14 for drinks/week for men and ≤7 drinks/week for women | | | |
|  | Yes | The number of drinks per week (multiplying the number of days per week that alcohol was consumed the number of drinks per day): > 14 for drinks/week for men and > 7 drinks/week for women | | | |

# Supplementary Figure 5. The distribution of frailty index in HRS, CHARLS, ELSA, and SHARE.


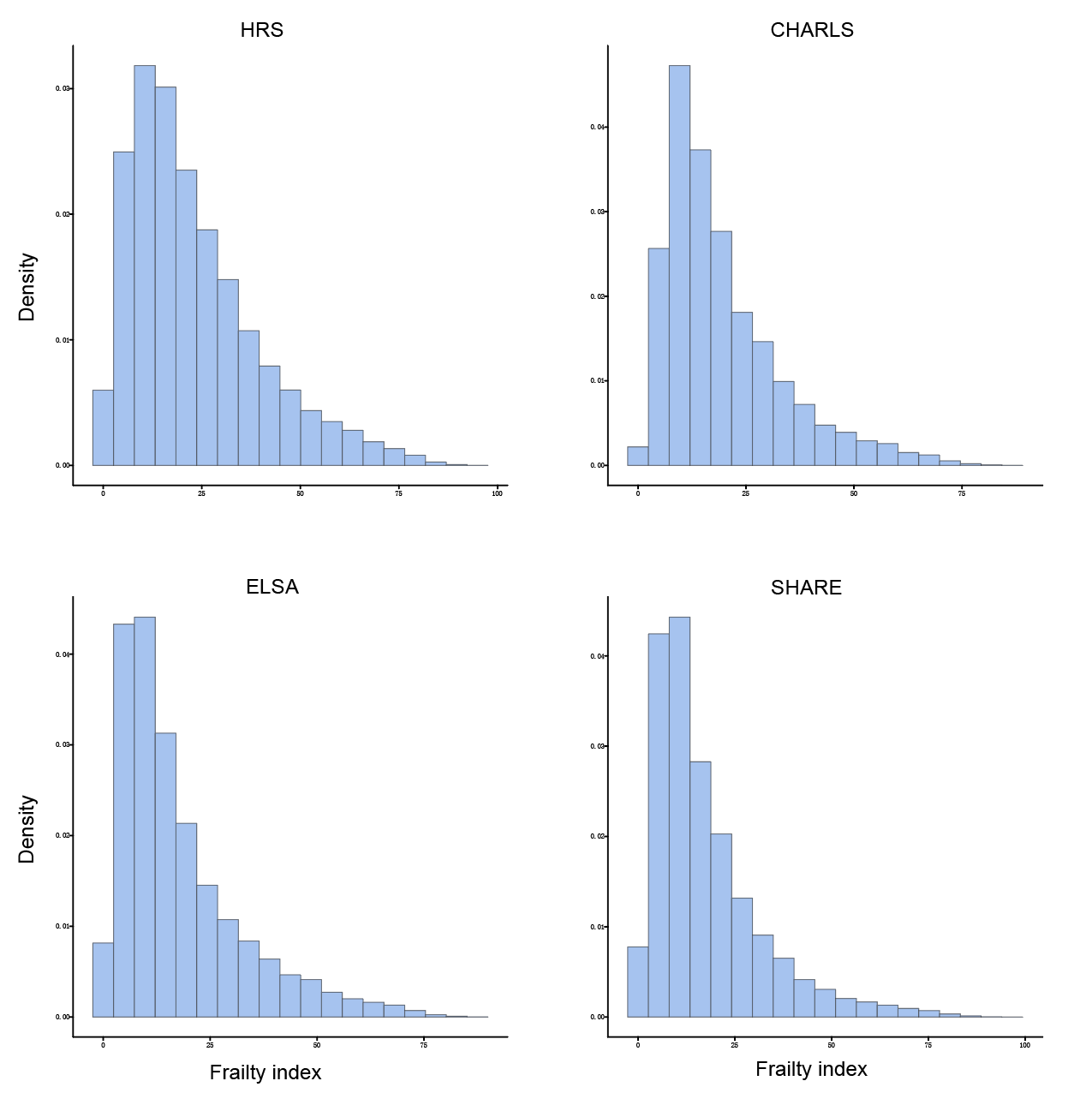


# Supplementary Table 5. Characteristics of participants by cardiometabolic diseases

|  | **No cardiometabolic diseases**  **(n=257,525)** | **Diabetes**  **(n=70,841)** | **Heart disease**  **(n= 92,838)** | **Stroke**  **(n= 27,912)** |
| --- | --- | --- | --- | --- |
| **Frailty** | 14.23 ± 11.23 | 29.28 ± 16.64 | 30.05 ± 16.78 | 37.66 ± 19.00 |
| **Age** | 64.56 ± 9.66 | 69.01 ± 9.92 | 71.66 ± 10.28 | 72.70 ± 10.57 |
| Missing | 207 | 25 | 42 | 19 |
| **Gender** |  |  |  |  |
| Female | 147,173 (57.1%) | 38,022 (53.7%) | 48,429 (52.2%) | 14,608 (52.3%) |
| Male | 110,350 (42.9%) | 32,819 (46.3%) | 44,409 (47.8%) | 13,304 (47.7%) |
| Missing | 2 | 0 | 0 | 0 |
| **Educational attainment** |  |  |  |  |
| Lower secondary  education or below | 103,142 (40.3%) | 30,475 (43.2%) | 40,952 (44.4%) | 12,278 (44.1%) |
| Upper secondary | 100,074 (39.1%) | 29,515 (41.8%) | 36,577 (39.6%) | 11,525 (41.4%) |
| Higher than upper  secondary | 52,626 (20.6%) | 10,568 (15.0%) | 14,738 (16.0%) | 4,009 (14.4%) |
| Missing | 1,683 | 283 | 571 | 100 |
| **Marital status** |  |  |  |  |
| Other | 81,658 (31.7%) | 27,540 (38.9%) | 36,051 (38.8%) | 12,473 (44.7%) |
| Married | 175,867 (68.3%) | 43,301 (61.1%) | 56,787 (61.2%) | 15,439 (55.3%) |
| **Obesity** |  |  |  |  |
| Underweight or normal (<25 kg/m^2^) | 100,340 (39.7%) | 14,120 (20.4%) | 27,905 (30.8%) | 8,991 (33.1%) |
| Overweight  (25-29.9 kg/m^2^) | 100,935 (39.9%) | 26,438 (38.3%) | 36,339 (40.1%) | 10,434 (38.4%) |
| Obesity (≥30 kg/m^2^) | 51,466 (20.4%) | 28,528 (41.3%) | 26,466 (29.1%) | 7,722 (28.4%) |
| Missing | 4,784 | 1,755 | 2,128 | 765 |
| **Hypertension** | 96,560 (37.9%) | 53,056 (75.0%) | 63,918 (69.0%) | 20,929 (75.1%) |
| Missing | 2,750 | 135 | 192 | 39 |
| **Cancer** | 20,524 (8.1%) | 8,895 (12.6%) | 12,915 (13.9%) | 4,164 (14.9%) |
| Missing | 2,924 | 169 | 234 | 45 |
| **Lung disease** | 18,585 (7.3%) | 9,705 (13.7%) | 16,393 (17.7%) | 5,001 (17.9%) |
| Missing | 2,875 | 159 | 225 | 50 |
| **Current drinking** | 42,032 (16.3%) | 6,219 (8.8%) | 9,981 (10.8%) | 2,587 (9.3%) |
| Missing | 219 | 23 | 32 | 5 |
| **Current smoking** | 62,898 (24.6%) | 13,812 (19.6%) | 18,743 (20.3%) | 6,230 (22.5%) |
| Missing | 1,420 | 394 | 491 | 171 |
| **Physical activity** | 211,229 (82.0%) | 45,525 (64.3%) | 60,586 (65.3%) | 15,211 (54.5%) |
| Missing | 5 | 0 | 1 | 1 |

# Supplementary Figure 6. Subgroup analyses of the association between cardiometabolic diseases and frailty.


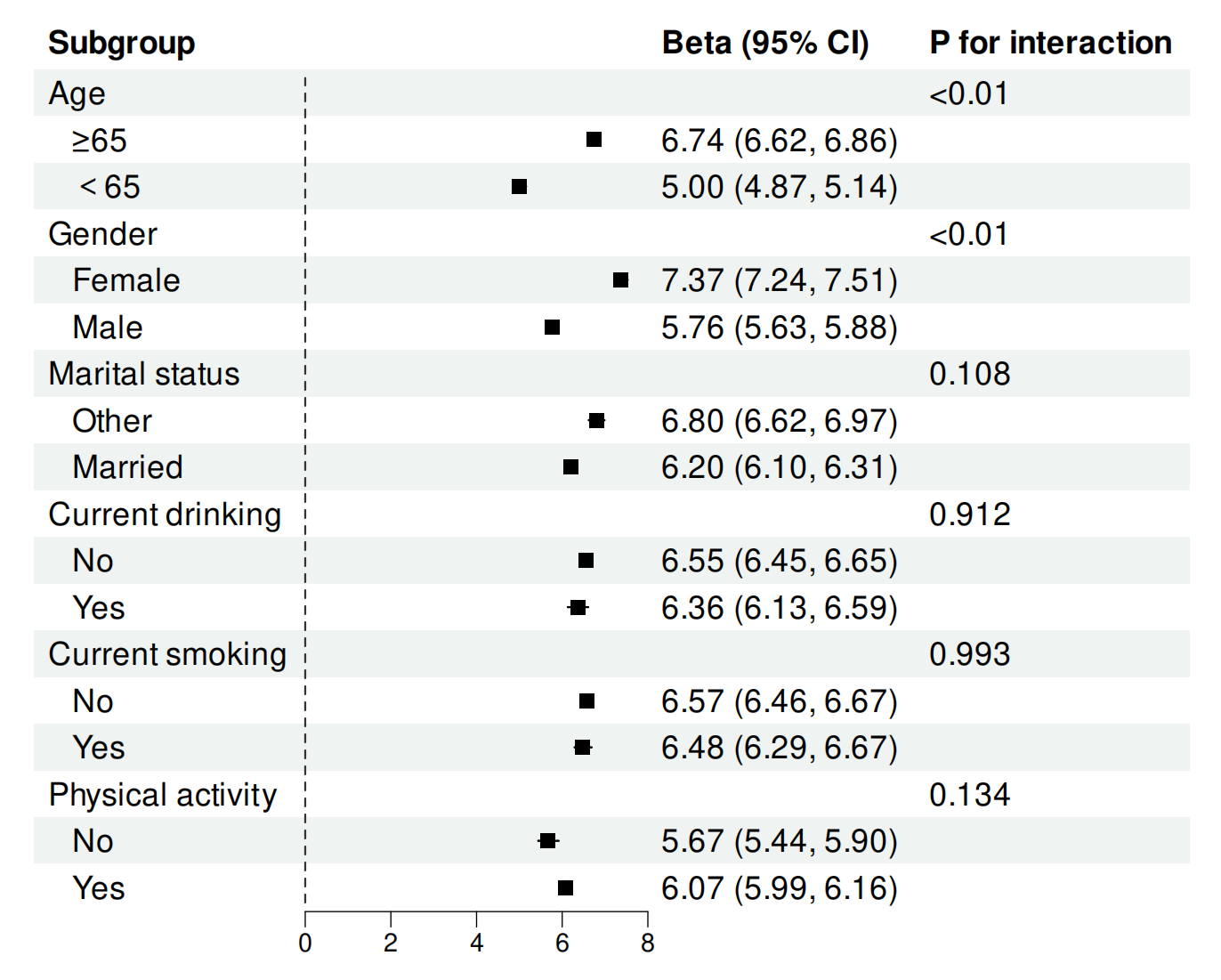


# Supplementary Table 6. Association between cardiometabolic diseases and frailty in HRS

| **Characteristic** | **Model 1** | | | **Model 2** | | | **Model 3** | | |
| --- | --- | --- | --- | --- | --- | --- | --- | --- | --- |
|  | **Beta** | **95% CI^1^** | **p-value** | **Beta** | **95% CI^1^** | **p-value** | **Beta** | **95% CI^1^** | **p-value** |
| **No cardiometabolic diseases** | Ref | | | Ref | | | Ref | | |
| **Diabetes** | 11.30 | 11.09, 11.51 | <0.001 | 10.76 | 10.57, 10.96 | <0.001 | 6.50 | 6.32, 6.67 | <0.001 |
| **Heart disease** | 14.61 | 14.41, 14.82 | <0.001 | 12.45 | 12.25, 12.66 | <0.001 | 8.56 | 8.39, 8.74 | <0.001 |
| **Stroke** | 19.35 | 19.03, 19.66 | <0.001 | 16.71 | 16.41, 17.01 | <0.001 | 12.01 | 11.76, 12.27 | <0.001 |
| **Diabetes+heart disease** | 18.67 | 18.35, 18.99 | <0.001 | 16.91 | 16.61, 17.22 | <0.001 | 11.09 | 10.83, 11.34 | <0.001 |
| **Diabetes+stroke** | 24.23 | 23.73, 24.74 | <0.001 | 22.03 | 21.56, 22.51 | <0.001 | 15.13 | 14.73, 15.53 | <0.001 |
| **Heart disease+stroke** | 23.85 | 23.42, 24.29 | <0.001 | 20.77 | 20.36, 21.18 | <0.001 | 14.69 | 14.34, 15.04 | <0.001 |
| **Diabetes+heart disease +stroke** | 28.21 | 27.56, 28.87 | <0.001 | 25.64 | 25.02, 26.26 | <0.001 | 17.61 | 17.10, 18.13 | <0.001 |
| ^1^CI = Confidence Interval | | | | | | | | | |
| Model 1 : no covariates were adjusted  Model 2 : adjusted for Age and Gender  Model 3 : adjusted for Age Gender, Marital status, Educational attainment, Obesity, Hypertension, Cancer, Lung disease, Current drinking, Current smoking, and Physical activity. | | | | | | | | | |

# Supplementary Table 7. Association between cardiometabolic diseases and frailty in CHARLS

| **Characteristic** | **Model 1** | | | **Model 2** | | | **Model 3** | | |
| --- | --- | --- | --- | --- | --- | --- | --- | --- | --- |
|  | **Beta** | **95% CI^1^** | **p-value** | **Beta** | **95% CI^1^** | **p-value** | **Beta** | **95% CI^1^** | **p-value** |
| **No cardiometabolic diseases** | Ref | | | Ref | | | Ref | | |
| **Diabetes** | 9.30 | 8.96, 9.65 | <0.001 | 8.46 | 8.13, 8.78 | <0.001 | 6.30 | 5.99, 6.61 | <0.001 |
| **Heart disease** | 11.04 | 10.77, 11.31 | <0.001 | 9.54 | 9.29, 9.80 | <0.001 | 7.03 | 6.78, 7.27 | <0.001 |
| **Stroke** | 18.47 | 17.99, 18.95 | <0.001 | 16.76 | 16.31, 17.22 | <0.001 | 13.91 | 13.48, 14.34 | <0.001 |
| **Diabetes+heart disease** | 15.97 | 15.39, 16.55 | <0.001 | 14.34 | 13.80, 14.89 | <0.001 | 10.62 | 10.11, 11.13 | <0.001 |
| **Diabetes+stroke** | 21.59 | 20.58, 22.60 | <0.001 | 19.82 | 18.87, 20.78 | <0.001 | 15.73 | 14.85, 16.61 | <0.001 |
| **Heart disease+stroke** | 22.95 | 22.12, 23.79 | <0.001 | 20.96 | 20.18, 21.75 | <0.001 | 16.63 | 15.90, 17.36 | <0.001 |
| **Diabetes+heart disease +stroke** | 24.66 | 23.20, 26.12 | <0.001 | 22.55 | 21.18, 23.92 | <0.001 | 17.55 | 16.28, 18.83 | <0.001 |
| ^1^CI = Confidence Interval | | | | | | | | | |
| Model 1 : no covariates were adjusted  Model 2 : adjusted for Age and Gender  Model 3 : adjusted for Age Gender, Marital status, Educational attainment, Obesity, Hypertension, Cancer, Lung disease, Current drinking, Current smoking, and Physical activity. | | | | | | | | | |

# Supplementary Table 8. Association between cardiometabolic diseases and frailty in ELSA

| **Characteristic** | **Model 1** | | | **Model 2** | | | **Model 3** | | |
| --- | --- | --- | --- | --- | --- | --- | --- | --- | --- |
|  | **Beta** | **95% CI^1^** | **p-value** | **Beta** | **95% CI^1^** | **p-value** | **Beta** | **95% CI^1^** | **p-value** |
| **No cardiometabolic diseases** | Ref | | | Ref | | | Ref | | |
| **Diabetes** | 11.82 | 11.31, 12.32 | <0.001 | 10.77 | 10.30, 11.24 | <0.001 | 5.95 | 5.54, 6.35 | <0.001 |
| **Heart disease** | 12.34 | 11.95, 12.72 | <0.001 | 10.26 | 9.88, 10.63 | <0.001 | 7.21 | 6.89, 7.52 | <0.001 |
| **Stroke** | 18.96 | 18.20, 19.72 | <0.001 | 15.61 | 14.88, 16.33 | <0.001 | 10.19 | 9.58, 10.79 | <0.001 |
| **Diabetes+heart disease** | 19.21 | 18.40, 20.02 | <0.001 | 17.28 | 16.52, 18.04 | <0.001 | 10.71 | 10.06, 11.35 | <0.001 |
| **Diabetes+stroke** | 25.16 | 23.61, 26.72 | <0.001 | 22.99 | 21.54, 24.44 | <0.001 | 14.57 | 13.38, 15.75 | <0.001 |
| **Heart disease+stroke** | 23.36 | 22.25, 24.47 | <0.001 | 19.90 | 18.85, 20.95 | <0.001 | 13.19 | 12.32, 14.06 | <0.001 |
| **Diabetes+heart disease +stroke** | 28.61 | 26.55, 30.68 | <0.001 | 26.41 | 24.48, 28.33 | <0.001 | 16.23 | 14.64, 17.81 | <0.001 |
| ^1^CI = Confidence Interval | | | | | | | | | |
| Model 1 : no covariates were adjusted  Model 2 : adjusted for Age and Gender  Model 3 : adjusted for Age Gender, Marital status, Educational attainment, Obesity, Hypertension, Cancer, Lung disease, Current drinking, Current smoking, and Physical activity. | | | | | | | | | |

# Supplementary Table 9. Association between cardiometabolic diseases and frailty in SHARE

| **Characteristic** | **Model 1** | | | **Model 2** | | | **Model 3** | | |
| --- | --- | --- | --- | --- | --- | --- | --- | --- | --- |
|  | **Beta** | **95% CI^1^** | **p-value** | **Beta** | **95% CI^1^** | **p-value** | **Beta** | **95% CI^1^** | **p-value** |
| **No cardiometabolic diseases** | Ref | | | Ref | | | Ref | | |
| **Diabetes** | 12.13 | 11.97, 12.29 | <0.001 | 10.21 | 10.06, 10.35 | <0.001 | 6.37 | 6.24, 6.49 | <0.001 |
| **Heart disease** | 14.28 | 14.15, 14.41 | <0.001 | 11.23 | 11.10, 11.36 | <0.001 | 7.84 | 7.73, 7.95 | <0.001 |
| **Stroke** | 19.43 | 19.20, 19.67 | <0.001 | 16.09 | 15.87, 16.30 | <0.001 | 11.87 | 11.69, 12.06 | <0.001 |
| **Diabetes+heart disease** | 19.40 | 19.16, 19.64 | <0.001 | 16.30 | 16.08, 16.52 | <0.001 | 10.76 | 10.57, 10.95 | <0.001 |
| **Diabetes+stroke** | 26.28 | 25.84, 26.71 | <0.001 | 22.69 | 22.30, 23.09 | <0.001 | 15.84 | 15.51, 16.18 | <0.001 |
| **Heart disease+stroke** | 22.32 | 22.04, 22.61 | <0.001 | 18.55 | 18.29, 18.81 | <0.001 | 13.65 | 13.43, 13.87 | <0.001 |
| **Diabetes+heart disease +stroke** | 28.86 | 28.36, 29.37 | <0.001 | 24.96 | 24.51, 25.42 | <0.001 | 17.56 | 17.17, 17.95 | <0.001 |
| ^1^CI = Confidence Interval | | | | | | | | | |
| Model 1 : no covariates were adjusted  Model 2 : adjusted for Age and Gender  Model 3 : adjusted for Age Gender, Marital status, Educational attainment, Obesity, Hypertension, Cancer, Lung disease, Current drinking, Current smoking, and Physical activity. | | | | | | | | | |

# Supplementary Table 10. Association between one cardiometabolic disease and frailty in HRS, CHARLS, ELSA, and SHARE

| **Characteristic** | **Model 1** | | | **Model 2** | | | **Model 3** | | |
| --- | --- | --- | --- | --- | --- | --- | --- | --- | --- |
|  | **Beta** | **95% CI^1^** | **p-value** | **Beta** | **95% CI^1^** | **p-value** | **Beta** | **95% CI^1^** | **p-value** |
| HRS | 6.51 | 6.31, 6.72 | <0.001 | 5.2 | 5.01, 5.39 | <0.001 | 3.72 | 3.55, 3.89 | <0.001 |
| CHARLS | 8.58 | 8.32, 8.83 | <0.001 | 7.33 | 7.10, 7.57 | <0.001 | 5.06 | 4.83, 5.29 | <0.001 |
| ELSA | 8.61 | 8.23, 8.99 | <0.001 | 6.73 | 6.36, 7.09 | <0.001 | 4.20 | 3.90, 4.51 | <0.001 |
| SHARE | 7.73 | 7.59, 7.87 | <0.001 | 5.20 | 5.07, 5.33 | <0.001 | 3.14 | 3.04, 3.25 | <0.001 |
| ^1^CI = Confidence Interval | | | | | | | | | |
| Model 1 : no covariates were adjusted  Model 2 : adjusted for Age and Gender  Model 3 : adjusted for Age Gender, Marital status, Educational attainment, Obesity, Hypertension, Cancer, Lung disease, Current drinking, Current smoking, and Physical activity. | | | | | | | | | |

# Supplementary Table 11. Association between two cardiometabolic disease and frailty in HRS, CHARLS, ELSA, and SHARE

| **Characteristic** | **Model 1** | | | **Model 2** | | | **Model 3** | | |
| --- | --- | --- | --- | --- | --- | --- | --- | --- | --- |
|  | **Beta** | **95% CI^1^** | **p-value** | **Beta** | **95% CI^1^** | **p-value** | **Beta** | **95% CI^1^** | **p-value** |
| HRS | 17.36 | 17.07, 17.65 | <0.001 | 15.31 | 15.03, 15.59 | <0.001 | 10.15 | 9.91, 10.38 | <0.001 |
| CHARLS | 17.09 | 16.60, 17.59 | <0.001 | 15.41 | 14.95, 15.88 | <0.001 | 11.72 | 11.28, 12.16 | <0.001 |
| ELSA | 19.05 | 18.35, 19.75 | <0.001 | 16.62 | 15.95, 17.28 | <0.001 | 10.8 | 10.24, 11.36 | <0.001 |
| SHARE | 17.88 | 17.67, 18.08 | <0.001 | 14.69 | 14.50, 14.88 | <0.001 | 10.03 | 9.87, 10.20 | <0.001 |
| ^1^CI = Confidence Interval | | | | | | | | | |
| Model 1 : no covariates were adjusted  Model 2 : adjusted for Age and Gender  Model 3 : adjusted for Age Gender, Marital status, Educational attainment, Obesity, Hypertension, Cancer, Lung disease, Current drinking, Current smoking, and Physical activity. | | | | | | | | | |

# Supplementary Table 12. Association between three cardiometabolic disease and frailty in HRS, CHARLS, ELSA, and SHARE

| **Characteristic** | **Model 1** | | | **Model 2** | | | **Model 3** | | |
| --- | --- | --- | --- | --- | --- | --- | --- | --- | --- |
|  | **Beta** | **95% CI^1^** | **p-value** | **Beta** | **95% CI^1^** | **p-value** | **Beta** | **95% CI^1^** | **p-value** |
| HRS | 28.21 | 27.56, 28.87 | <0.001 | 25.64 | 25.02, 26.26 | <0.001 | 17.61 | 17.10, 18.13 | <0.001 |
| CHARLS | 24.66 | 23.20, 26.12 | <0.001 | 22.55 | 21.18, 23.92 | <0.001 | 17.55 | 16.28, 18.83 | <0.001 |
| ELSA | 28.61 | 26.55, 30.68 | <0.001 | 26.41 | 24.48, 28.33 | <0.001 | 16.23 | 14.64, 17.81 | <0.001 |
| SHARE | 28.86 | 28.36, 29.37 | <0.001 | 24.96 | 24.51, 25.42 | <0.001 | 17.56 | 17.17, 17.95 | <0.001 |
| ^1^CI = Confidence Interval | | | | | | | | | |
| Model 1 : no covariates were adjusted  Model 2 : adjusted for Age and Gender  Model 3 : adjusted for Age Gender, Marital status, Educational attainment, Obesity, Hypertension, Cancer, Lung disease, Current drinking, Current smoking, and Physical activity. | | | | | | | | | |
